# Complete Pneumothorax Secondary to a Ruptured Pulmonary Hydatid Cyst: A Case Report and Brief Review of the Literature

**DOI:** 10.7759/cureus.116838

**Published:** 2026-09-24

**Authors:** Houda Snene, Monia Attia, Khaoula Ben Amor, Nadia Mehiri, Bechir Louzir

**Affiliations:** 1 Department of Pulmonology and Allergology, Mongi Slim University Hospital, Tunis El Manar University, La Marsa, TUN; 2 Department of Medical Imaging, Mongi Slim University Hospital, Tunis El Manar University, La Marsa, TUN

**Keywords:** case report, echinococcus granulosus, literature review, pneumothorax, pulmonary hydatid cyst

## Abstract

Pulmonary hydatid disease, caused by *Echinococcus granulosus*, remains a major public health concern in endemic regions. While simple cysts are often asymptomatic or present with mild respiratory symptoms, cyst rupture into the pleural cavity leading to a complete pneumothorax represents a rare, life-threatening acute complication. A 19-year-old woman with a two-month history of progressive exertional dyspnea presented to the emergency department with acute right-sided chest pain of 48 hours' duration. Physical examination showed no acute respiratory distress, and laboratory tests revealed no systemic inflammatory syndrome. Chest radiography demonstrated a right hilar opacity of water density with a rectilinear air-fluid level, associated with complete right lung collapse consistent with a total pneumothorax. Chest computed tomography (CT) confirmed a cystic lesion containing a partially collapsed membrane, characteristic of a ruptured pulmonary hydatid cyst causing a pneumothorax. Initial management consisted of emergency chest tube thoracostomy and broad-spectrum empirical antibiotic therapy, followed by definitive surgical resection. Ruptured pulmonary hydatid cyst must be systematically included in the differential diagnosis of acute pneumothorax with cavitary or fluid-containing lung opacities in endemic areas. Emergency chest CT and a combined medico-surgical approach are critical for successful management.

## Introduction

Echinococcosis is a widespread zoonotic infection caused by the larval stage of *Echinococcus granulosus *[1]. It remains highly endemic in rural and pastoral regions of North Africa, Southern Europe, Central Asia, and South America, where close contact between humans, sheep, and dogs is prevalent [2,3]. The lung is the second most common site of involvement after the liver, accounting for 20% to 30% of cases [1,2]. Intact pulmonary hydatid cysts often remain asymptomatic for long periods or manifest with non-specific symptoms such as chronic cough, chest pain, or dyspnea [2,3]. Complications arise primarily when the cyst enlarges and ruptures into adjacent anatomical structures [2,4]. Rupture into the tracheobronchial tree is relatively common, but erosion into the pleural cavity causing an acute total or complete pneumothorax is a rare emergency, occurring in less than 5% of complicated cases [4,5]. Diagnosing intrapleural cyst rupture can be particularly challenging when initial radiographic features mimic other conditions, such as a lung abscess or pulmonary tuberculosis, owing to atypical rectilinear air-fluid levels rather than classic floating membrane signs [3,6]. Here, we report a case of complete right pneumothorax secondary to a ruptured pulmonary hydatid cyst in a young female patient and provide a brief literature review highlighting diagnostic nuances and management strategies.

## Case presentation

A 19-year-old woman presented to the emergency department complaining of sudden right-sided pleuritic chest pain that had developed 48 hours prior. She reported a history of exertional dyspnea that had been gradually worsening over the preceding two months. She had no associated fever, hemoptysis, or systemic complaints. Epidemiologically, she resided in a semi-rural agricultural region with frequent contact with livestock and free-roaming farm dogs. On physical examination, she was eupneic with a peripheral oxygen saturation of 98% on room air. Vital signs were completely normal: blood pressure 120/70 mmHg, heart rate 88 beats/minute, respiratory rate 24 breaths/minute, and body temperature 36.5°C. Thoracic auscultation revealed absent breath sounds over the right lung field. Laboratory testing demonstrated no evidence of a biological inflammatory response: white blood cell count was 9340/µL (with hypereosinophilia of 1570/µL) and C-reactive protein was 16 mg/L. Chest radiography (Figure 1) demonstrated a right hilar opacity of water density featuring a sharp, rectilinear air-fluid level (red arrow in Figure 1). This lesion was surrounded by a massive peripheral avascular hyperclarity, indicating a large right pneumothorax with severe lung collapse.

**Figure 1:**
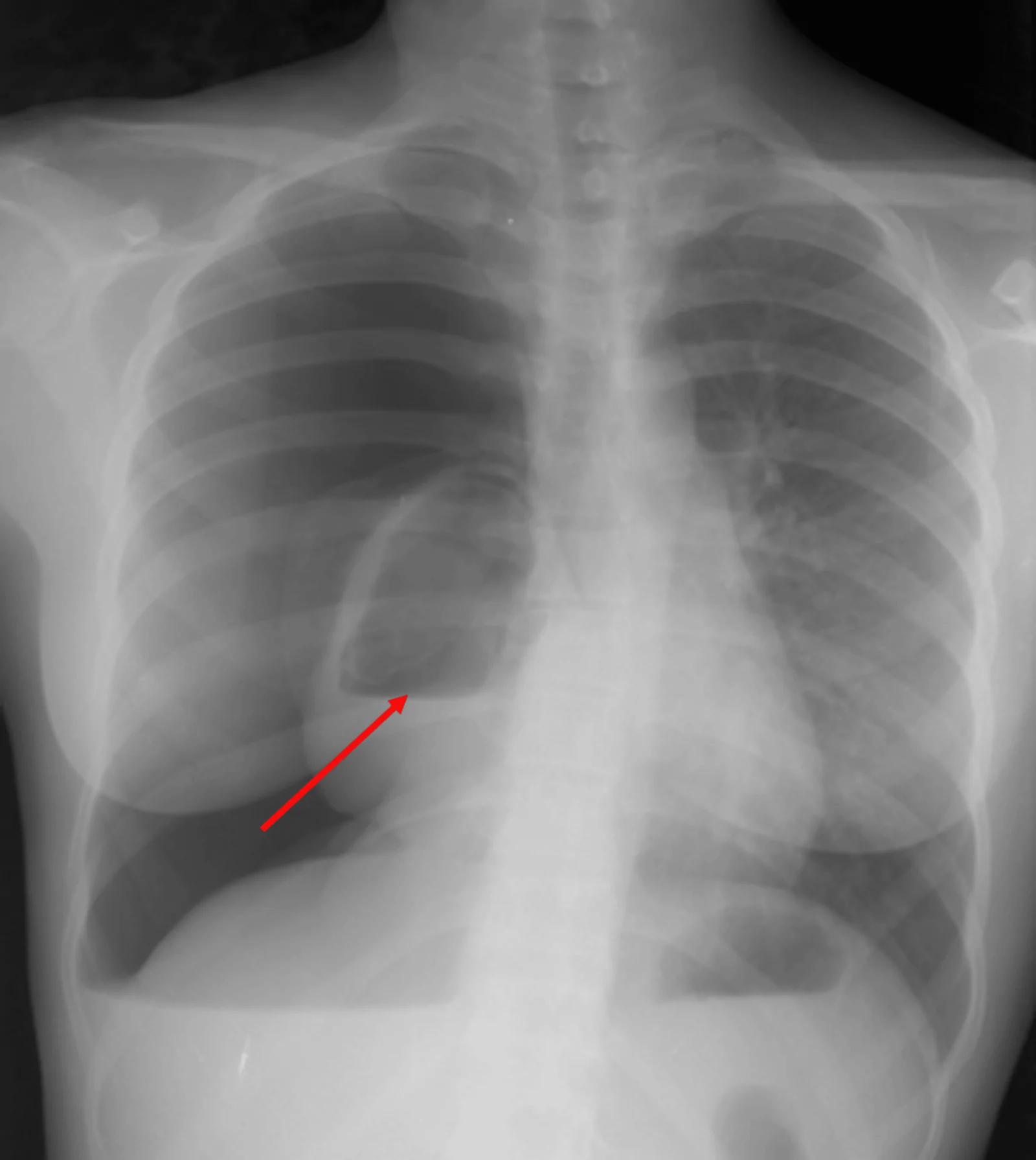
Chest X-ray revealing right hilar opacity of water density with an air-fluid level (red arrow) associated with peripheral avascular hyperclarity consistent with a large right pneumothorax. Note that the air-fluid level is straight rather than wavy, which can initially mimic a lung abscess.

To further characterize the underlying etiology, contrast-enhanced chest computed tomography (CT) was performed. Coronal reconstruction in the parenchymal window (Figure 2) revealed a well-defined cystic lesion containing a partially collapsed internal membrane (the ‘camalote’ or ‘water lily’ sign appearance: red arrows in Figure 2), directly communicating with the pleural space. These findings confirmed a filled, complicated pulmonary hydatid cyst associated with a complete secondary pneumothorax.

**Figure 2:**
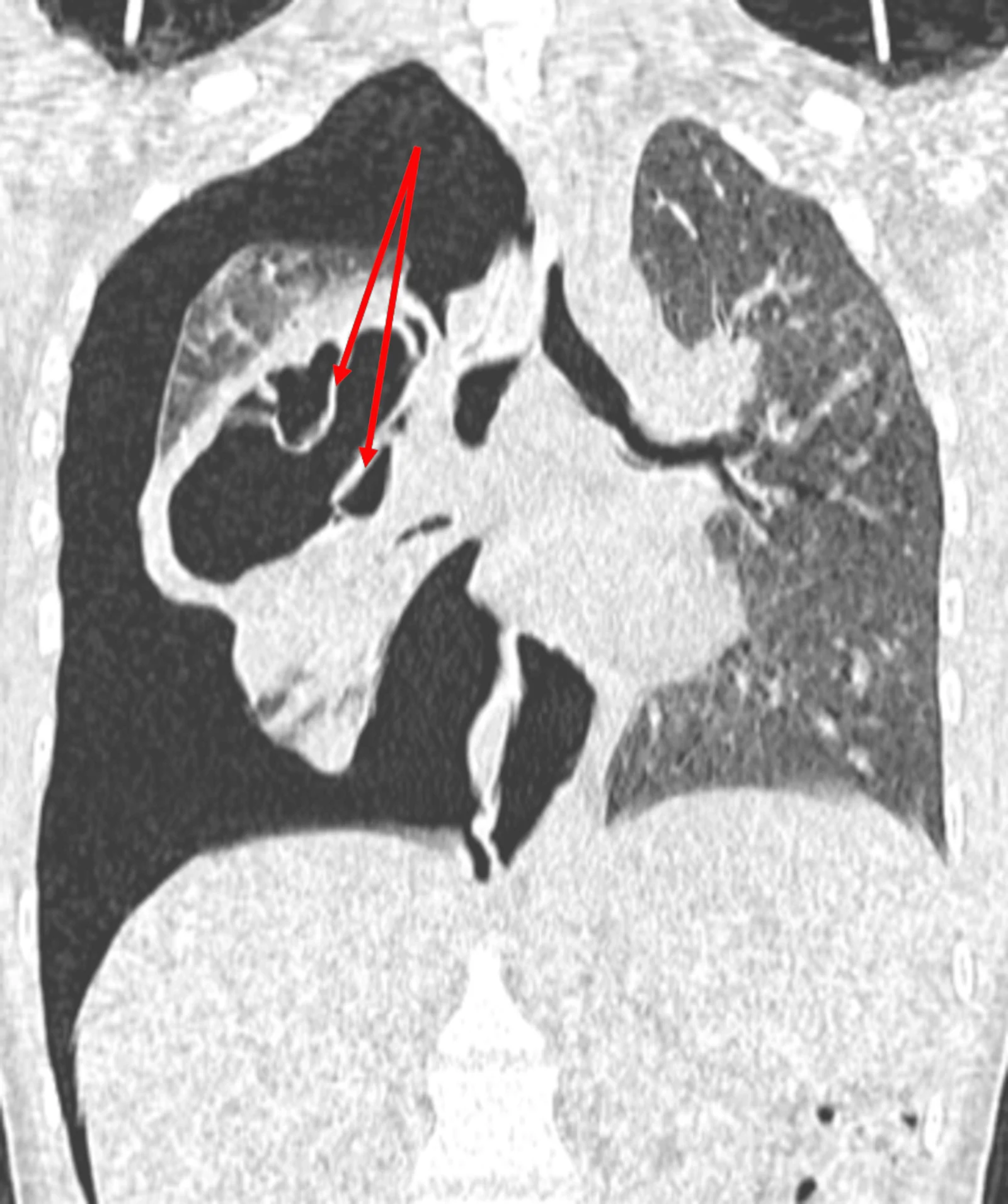
Coronal section in the parenchymal window of a chest CT scan revealing a cystic lesion containing a partially collapsed membrane (red arrows) suggestive of a filled hydatid cyst of the lung complicated by a pneumothorax. The partially collapsed membrane confirms intrapleural rupture of the cyst.

To rule out concomitant extrapulmonary disease, particularly hepatic hydatidosis, which coexists in 10% to 25% of cases, CT scans of the liver were performed, confirming the absence of liver or abdominal cysts. Emergency management involved the placement of a right intercostal chest drain, which led to significant evacuation of air and partial lung re-expansion. The patient was started on empirical broad-spectrum intravenous antibiotics (amoxicillin-clavulanic acid and ciprofloxacin) to prevent secondary pleural empyema given the broncho-pleural communication. Following stabilization, she underwent definitive surgical intervention (cystectomy with closure of bronchial communications and capitonnage). The postoperative course was uneventful, and no recurrence was noted at on year follow-up. The follow-up chest X-ray (Figure 3) six months after surgery shows no pulmonary recurrence.

**Figure 3:**
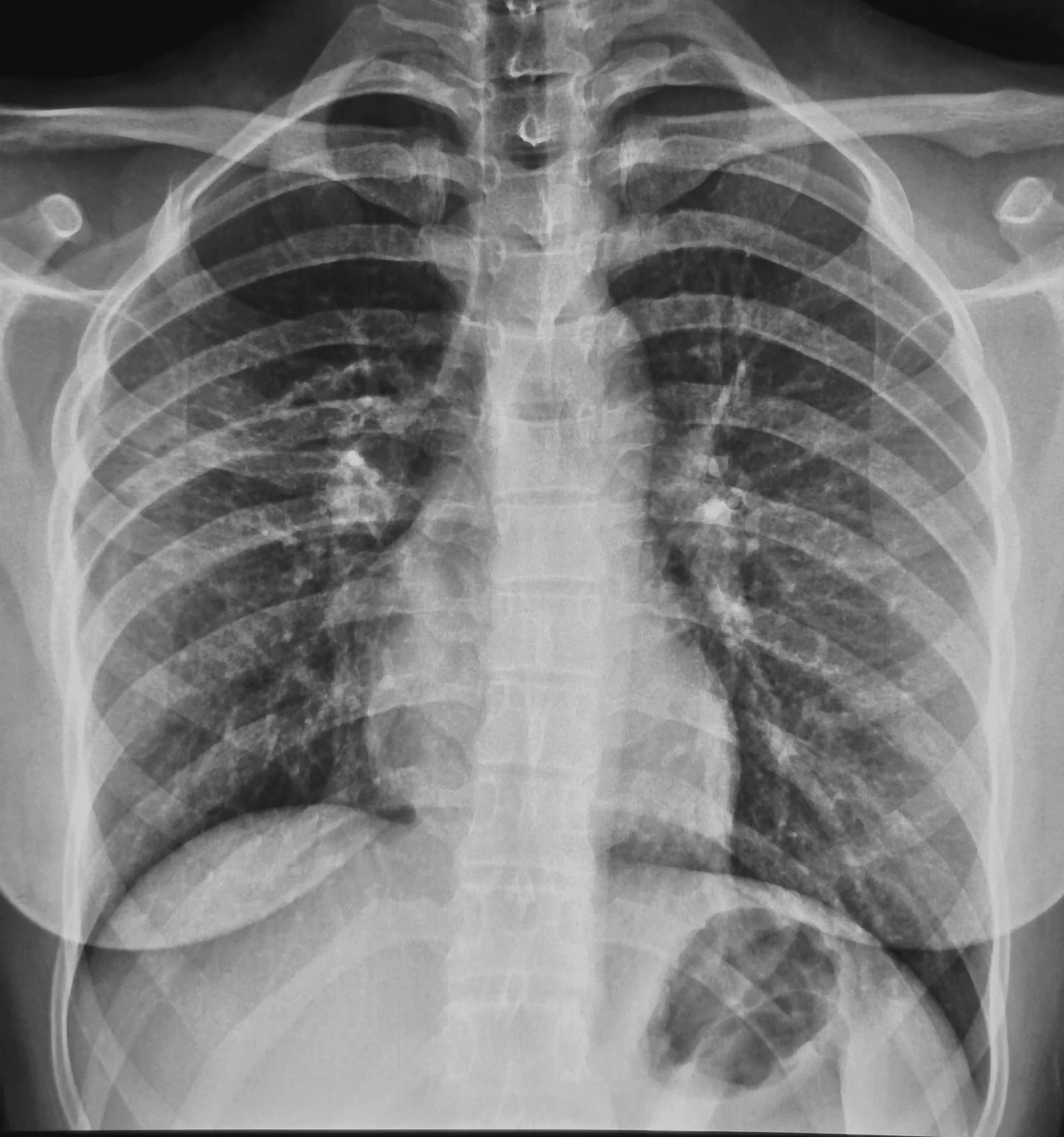
Chest X-ray at six-month follow-up showing no pulmonary recurrence.

## Discussion

Spontaneous rupture of a pulmonary hydatid cyst into the pleural cavity is a major thoracic emergency [4,5,7]. While intra-bronchial rupture often leads to coughing up hydatid fluid and membrane fragments (hydatidoptysis), intra-pleural erosion creates a direct communication between the bronchial tree, the cyst cavity, and the pleural space, resulting in pneumothorax or hydro-pneumothorax [4,6,7]. Pulmonary hydatid cysts grow progressively within the compliant lung parenchyma [2]. Rupture into the pleural cavity occurs when intracystic pressure exceeds the structural integrity of the pericyst, often triggered by violent coughing, minor trauma, or spontaneous tissue erosion [4,5,7]. This rupture leads to sudden decompression, pneumothorax, and varying degrees of pleural effusion or anaphylaxis due to the spillage of antigenic hydatid fluid [4,7]. Although the liver is the primary filter for Echinococcus oncospheres, pulmonary localization occurs when larvae bypass the hepatic sinusoids or travel via lymphatic pathways [1,8]. Concomitant hepatothoracic or extrapulmonary cysts are reported in 10-25% of patients [2,8]. Systematic abdominal ultrasonography is therefore mandatory in all patients presenting with pulmonary hydatidosis to rule out hepatic involvement [2,8]. In our patient, CT scans of the liver performed concurrently with the chest CT ruled out hepatic involvement. Serological testing (enzyme-linked immunosorbent assay (ELISA) or Western blot) has limited sensitivity in isolated pulmonary hydatid disease (30% to 60% false negatives) due to weak systemic immune stimulation when cyst membranes remain intact or isolated [3,8,9]. A negative serology should never exclude hydatid disease when radiological features are suggestive [3,8,9]. In our patient, it was not performed because the imaging was highly suggestive of pulmonary hydatid disease and the test would not have contributed significantly to the diagnosis.

Radiologically, the classical presentation of a ruptured pulmonary hydatid cyst on chest X-ray is a water-density opacity exhibiting a scalloped or wavy air-fluid level (the ‘water lily’ or ‘camalote’ sign), caused by the detached endocyst floating on top of residual fluid [6]. However, as illustrated in our patient's initial X-ray (Figure 1), the fluid level can sometimes appear rectilinear (red arrow in Figure 1) rather than scalloped, mimicking a classic lung abscess or infected cavitary lesion [3,6]. This occurs when the endocyst is only partially collapsed (red arrows in Figure 2) or trapped against the cyst wall, as confirmed on the coronal CT section (Figure 2) [3,6].

In young patients presenting with a cavitary/cystic pulmonary opacity and secondary pneumothorax, three principal differential diagnoses must be considered [3]. Lung abscess complicated by pneumothorax is characterized by thick, irregular walls with a typical rectilinear air-fluid level, accompanied by marked systemic inflammatory signs and purulent sputum [3,6]. Pulmonary tuberculosis complicated by pneumothorax commonly presents with cavitary lesions in the apical/posterior segments, mediastinal lymphadenopathy, and significant constitutional symptoms including asthenia, anorexia, and weight loss [3,6]. Bronchopulmonary carcinoma complicated by pneumothorax involves necrotic cavitated tumors eroding into the pleura, typically seen in older patients or smokers [3,6].

A targeted narrative literature review was conducted across PubMed/MEDLINE to select representative clinical series evaluating intrapleural rupture and secondary pneumothorax caused by pulmonary hydatid cysts, focusing on studies with comprehensive surgical and outcome data (Table 1) and particulary whom with similarity to the characteristics of our study population.

**Table 1:** Summary of literature series and current case of pneumothorax secondary to ruptured pulmonary hydatid cyst.

Study/Author	Design/Sample Size	Patient Characteristics	Key Clinical/Radiological Features	Therapeutic Approach	Outcomes
Current case	Single case report	19/F	Acute chest pain, chronic exertional dyspnea; complete pneumothorax with hilar cyst and partially collapsed membrane	Emergency chest tube drainage + empirical antibiotics + delayed cystectomy and capitonnage	Complete recovery without recurrence
Kuzucu et al. [4]	Retrospective series (N = 67 total/33 complicated)	Mean age: 28.5 years (range 5-72 yrs)	Expectorated cyst contents, hemoptysis, fever; pleural effusion/hydro-pneumothorax	Emergency/elective surgery (cystectomy + capitonnage) + albendazole	Favorable outcome, primary surgical cure
Aribas et al. [5]	Retrospective series (N = 43 with pleural complications)	Mean age: 30 years; M/F ratio: 1.05 (22M/21F)	Spontaneous pneumothorax (6.2%), empyema, hydro-pneumothorax; chest pain, fever	Tube thoracostomy followed by thoracotomy (cystectomy + capitonnage/decortication)	Favorable outcome (morbidity 16.3%, no recurrence)
Bouchikh et al. [7]	Retrospective series (N = 75)	Mean age: 23 years; male predominance	Sudden chest pain, dyspnea, acute pleural effusion/hydro-pneumothorax; anaphylactic reactions (~12%)	Emergency thoracic drainage, conservative surgery (cystectomy + capitonnage/fistula closure)	Favorable overall outcome, low morbidity, no recurrence

The management of a hydatid cyst complicated by pneumothorax requires an urgent, staged approach [4,7]. The immediate phase involves intercostal chest drain insertion to relieve pleural tension and re-expand the collapsed lung, alongside broad-spectrum antibiotics to cover secondary bacterial superinfections [3,5,7]. The definitive phase involves surgical intervention (sparing lung tissue via cystectomy, bronchial fistula closure, and pericystectomy or capitonnage) remains the gold standard to prevent recurrence, chronic broncho-pleural fistulas, or pleural empyema [4,7]. Perioperative antiparasitic treatment (albendazole) is routinely administered to minimize the risk of secondary dissemination [4].

## Conclusions

Ruptured pulmonary hydatid cyst is a rare but critical cause of secondary complete pneumothorax in endemic countries. The presence of an atypical rectilinear fluid level on chest radiography should not rule out hydatid disease. Early chest CT scanning is crucial to visualize collapsed membranes, allowing for prompt intercostal drainage and surgical cure. Abdominal ultrasonography or CT scans of the liver should be systematically routinely performed to exclude coexisting liver involvement. Clinicians should maintain a high index of suspicion when evaluating non-resolving pneumothorax associated with cavitary opacities in high-prevalence settings. A multidisciplinary management plan combining immediate drainage, targeted medical treatment, and radical surgical intervention is fundamental to prevent severe pleuropulmonary sequelae.
